# HER3 over-expression and overall survival in gastrointestinal cancers

**DOI:** 10.18632/oncotarget.5998

**Published:** 2015-10-17

**Authors:** Yadong Wang, Haiyan Yang, Guangcai Duan

**Affiliations:** ^1^ Department of Toxicology, Henan Center for Disease Control and Prevention, Zhengzhou 450016, China; ^2^ Henan Collaborative Innovation Center of Molecular Diagnosis and Laboratory Medicine, Xinxiang Medical University, Xinxiang 453003, China; ^3^ Department of Epidemiology, School of Public Health, Zhengzhou University, Zhengzhou 450001, China

**Keywords:** HER3, gastrointestinal cancers, overall survival

## Abstract

Published studies on the association between human epidermal factor receptor 3 (HER3) expression and overall survival (OS) in gastrointestinal cancers have yielded conflicting results. The aim of this study was to explore the association of HER3 over-expression with OS in gastrointestinal cancers. A systematic search was performed through Medline/PubMed, Embase, Science Direct and Elsevier. The summary odds ratio (OR) with 95% confidence interval (CI) was calculated to estimate the strength of the association. Overall, we observed that HER3 over-expression was associated with worse OS at five years (OR = 1.38, 95% CI: 1.04–1.82); however, HER3 over-expression was not associated with worse OS at three years (OR = 1.33, 95% CI: 0.97–1.84). The cumulative meta-analysis showed similar results. In subgroup analyses by tumor type, HER3 over-expression in gastric cancers was associated with worse OS at both three years (OR = 1.69, 95% CI: 1.28–2.25) and five years (OR = 1.74, 95% CI: 1.26–2.41). In conclusion, our results suggest that HER3 over-expression may be associated with worse overall survival in gastric cancers. Well-designed studies with a large sample size are required to further confirm our findings.

## INTRODUCTION

Gastrointestinal cancers are a group of highly aggressive malignancies (primarily including gastric carcinoma and colorectal cancer) that constitute a major public health problem worldwide. In 2015 in the United States of America alone, 291,000 new cases are estimated to be diagnosed, and approximately 149,000 patients will die from gastrointestinal cancers [1]. However, the combination of surgery, radiotherapy and chemotherapy remains the standard treatment for gastrointestinal cancer cases, but not all patients derive a benefit from it. Therefore, it is of great clinical value to identify applicable prognostic biomarkers that may not only improve a poor prognosis but also provide novel therapeutic targets.

HER3 (ErbB3) is a member of the human epidermal growth factor receptor (EGFR) family of receptor tyrosine kinases, which consists of four members: HER1/ErbB1, HER2/ErbB2, HER3/ErbB3 and HER4/ErbB4 [2]. Among the ErbB family, HER3 is a unique member because it lacks intrinsic tyrosine kinase activity and can't form a homodimer; Thus, HER3 forms heterodimers with other members of the ErbB family to carry out its role in signal transduction [3]. HER3 could effectively couple to the PI3K/AKT pathway, thereby controlling different biological outcomes, including cell proliferation, motility and cell survival [4]. Studies have shown that HER3 plays a key role in the pathogenesis of various human solid tumors [5]. The over-expression of HER3 has been illustrated in various cancers, including stomach cancer and colorectal cancer [6]. Ciardiello et al. reported that HER3 mRNA was detected in 55% of primary or metastatic human colorectal carcinomas but in only 22% of normal colon mucosa and 32% of normal liver samples [7]. Zhang et al.'s study showed that HER3 over-expression was detected in 14 (13.7%) of 102 gastric cancer patients and in 2 (2.0%) of a non-tumorous group of 102 specimens (13.7% vs. 2.0%, *P* < 0.01) [8]. Wu et al.'s study showed that HER3 over-expression was significantly increased in human gastric cancer compared with adjacent normal gastric tissues, as observed by both quantitative real-time reverse transcription-polymerase chain reaction (RT-PCR) and immunohistochemistry (IHC) [9].

The prognostic value and association with clinicopathologic parameters of HER3 expression have recently been investigated in a large series of patients with gastrointestinal cancers [3, 4, 9–17]. However, the results remain inconsistent and conflictive. To address this issue, we performed a meta-analysis to evaluate the potential role of HER3 in relation to overall survival in gastrointestinal cancers.

## RESULTS

### Study description

Among eleven studies, five evaluated colorectal cancer and six evaluated gastric cancer. A total of 1,963 patients was included in this study. All eleven studies reported data that allowed calculation of the overall survival at three years. Ten studies reported data that allowed calculation of the overall survival at five years.

### Test of heterogeneity

Heterogeneity was analyzed for the eleven eligible studies. Our results indicated the presence of heterogeneity in all analyses (Table 1). Therefore, we calculated the pooled ORs for the analyses using a random-effects model.

**Table 1:** Summary odds ratio of the association between HER3 over-expression and overall survival (OS) in gastrointestinal cancers

Studies	Number of studies	Heterogeneity test		Analysis model	OR (95% CI)	Hypothesis test		Begg's test		Egger's test	
		*Q*	*P*			*Z*	*P*	*Z*	*P*	*t*	*P*
Overall											
3-year OS	11	40.12	0.000	Random-effects model	1.33 (0.97–1.84)	1.74	0.081	0.31	0.755	0.59	0.572
5-year OS	10	41.72	0.000	Random-effects model	1.38 (1.04–1.82)	2.26	0.024	1.25	0.210	0.96	0.364
Gastric cancer											
3-year OS	6	11.40	0.044	Random-effects model	1.69 (1.28–2.25)	3.66	0.000	0.38	0.707	1.18	0.302
5-year OS	5	15.67	0.003	Random-effects model	1.74 (1.26–2.41)	3.32	0.001	2.20	0.027	7.28	0.005
Colorectal cancer											
3-year OS	5	12.34	0.015	Random-effects model	0.84 (0.48–1.47)	0.62	0.536	0.73	0.462	0.46	0.679
5-year OS	5	17.38	0.002	Random-effects model	1.05 (0.67–1.65)	0.20	0.840	0.73	0.462	0.57	0.608

### Quantitative data synthesis

**Figure 1:**
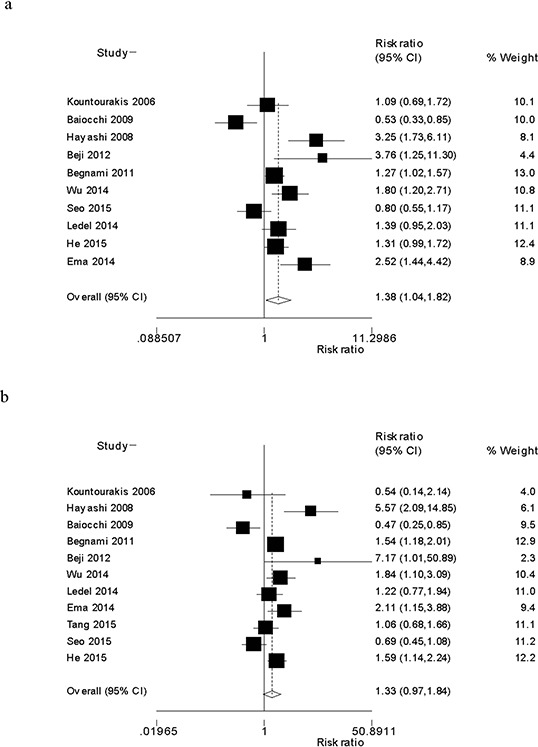
**Forest plot of the odds ratio of the association between HER3 over-expression and overall survival at five years a.** and three years **b.**

Table 1 lists the summary ORs of the association between HER3 over-expression and overall survival in gastrointestinal cancers. Overall, HER3 over-expression was significantly associated with worse OS at five years (OR = 1.38, 95% CI: 1.04–1.82) (Fig. 1a). However, we did not observe an association between HER3 over-expression and worse OS at three years (OR = 1.33, 95% CI: 0.97–1.84) (Fig. 1b). In subgroup analyses by tumor type, HER3 over-expression in gastric cancers was associated with worse OS at both three years (OR = 1.69, 95% CI: 1.28–2.25) and five years (OR = 1.74, 95% CI: 1.26–2.41) (Table 1). However, we did not observe an association between HER3 over-expression and overall survival at three years and five years in colorectal cancers; the summary ORs were 0.84 (95% CI: 0.48–1.47) and 1.05 (95% CI: 0.67–1.65), respectively (Table 1).

**Figure 2:**
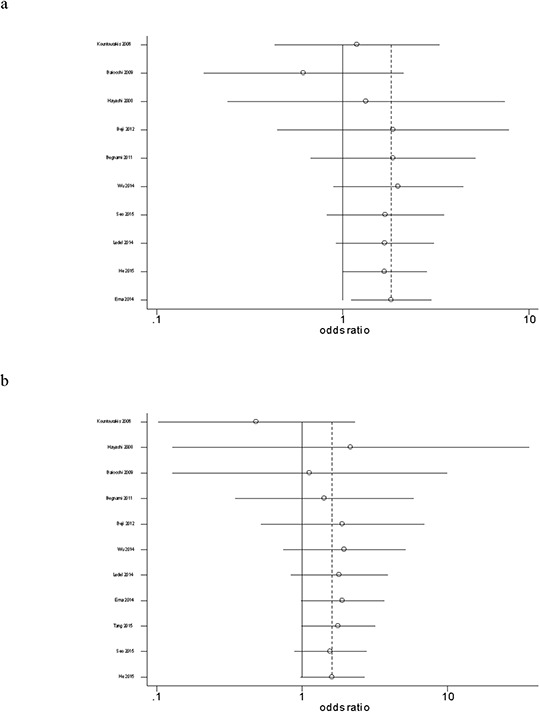
**Forest plot of the cumulative odds ratio of the association between HER3 over-expression and overall survival at five years a.** and three years **b.**

**Figure 3:**
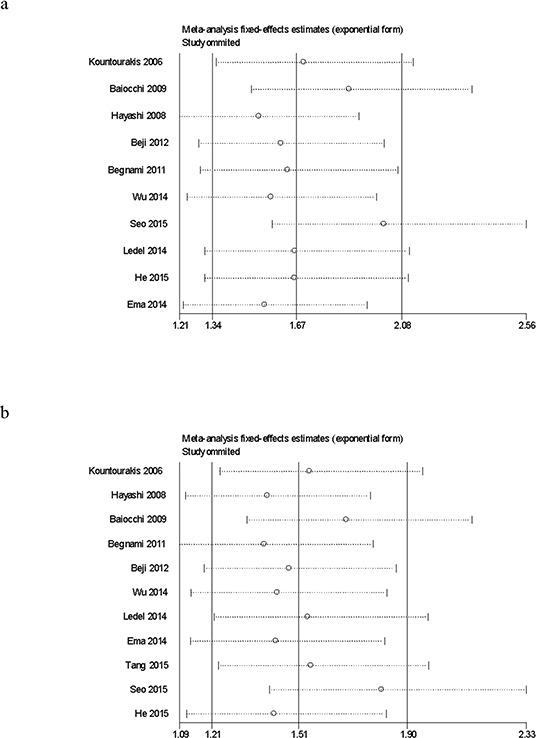
**Sensitivity analysis of the association between HER3 over-expression and overall survival at five years a.** and three years **b.**

The cumulative meta-analysis accumulated studies according to publication year and indicated that there was a significant association between HER3 over-expression and overall survival at five years among the total studies, and the cumulative OR was 1.82 (95% CI: 1.11–2.99) (Fig. 2a). Similarly, we did not observe a significant association between HER3 over-expression and overall survival at three years among the total studies, and the cumulative OR was 1.62 (95% CI: 0.98–2.68) (Fig. 2b). The sensitivity analysis showed that the results were robust and were not influenced by any single study (Figs. 3a and 3b).

### Bias diagnosis

**Figure 4:**
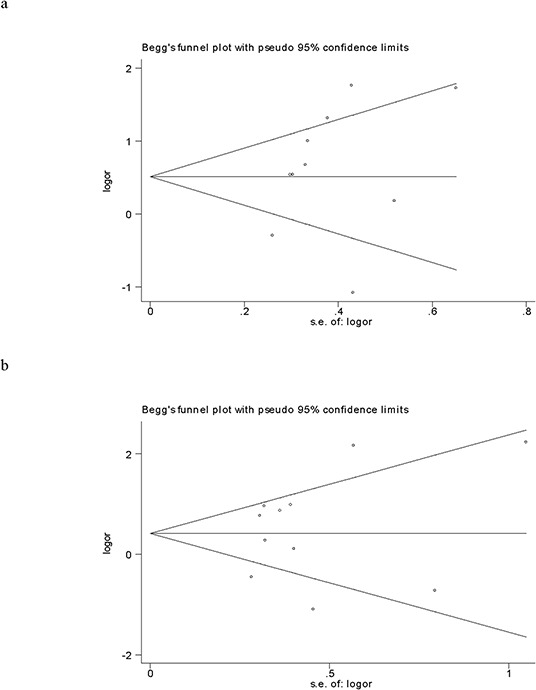
**Funnel plot analysis to detect publication bias for the association between HER3 over-expression and overall survival at five years a.** and three years **b.**

The shape of the funnel plots did not reveal any evidence of obvious asymmetry among the overall analysis (Figs. 4a and 4b), suggesting that there was no potential publication bias. Begg's test and Egger's test showed that there was no obvious publication bias in this study, except for the subgroup analysis of gastric cancers at five years overall survival, since the *P* value was 0.027 at Begg's test and 0.005 at Egger's test, respectively (Table 1).

## DISCUSSION

The HER3 gene is mapped to human chromosome 12q13 and encodes a 160-KDa transmembrane glycoprotein. HER3 has no functional kinase domain and requires dimerization with another receptor to activate downstream signal transduction pathways [10]. It plays an important role in cell proliferation and survival. Over-expression of HER3 has been reported in primary cancers and in cultured cells, including colon cancer and stomach cancer [6]. In this study, we conducted a meta-analysis of the published data concerning HER3 expression in gastrointestinal cancers and its relationship with overall survival, as determined through studies that detected HER3 by IHC. Our results indicated that HER3 over-expression was significantly associated with worse overall survival in gastrointestinal cancers at five years. Further cumulative meta-analysis demonstrated similar results. In the subgroup analysis stratified by tumor type, over-expression of HER3 was significantly associated with worse overall survival in gastric cancers at both three years and five years.

To the best of our knowledge, only one meta-analysis has reported an association between HER3 over-expression and survival in solid tumors [18]. The authors found that HER3 over-expression was associated with worse survival at both three years and five years in gastric cancers, but their study was based on only two studies with 325 patients, resulting in the enrolled studies and sample size being relatively small. Our results are consistent with those reported by Ocana et al. [18]. However, compared with the Ocana et al.'s study [18], our study has several advantages. First, we amplified the number of studies to six and enlarged the sample size to 1,038, making our findings more powerful and substantial. Second, a cumulative meta-analysis was performed to consolidate our findings. Third, a sensitivity analysis was conducted to check the effects of an individual study on the summary odds ratio. In addition, publication bias was assessed using the funnel plot, Begg's test and Egger's test. Ocana et al.'s study [18] and ours did not observe a significant association between HER3 over-expression and worse overall survival at three years and five years in colorectal cancers.

Although the underlying mechanisms involved in the association between HER3 over-expression and gastrointestinal cancers remain uncertain, several studies may allude the critical involvement of HER3 in the progression of gastrointestinal cancers to some extent. Lee et al. reported that HER3 knockdown induced cell cycle arrest and activation of Bak- and Bax-dependent apoptosis in colon cancer cells [19]. Beji et al.'s study showed that HER3 knockdown by RNA interference and anti-HER3 monoclonal antibody resulted in the inhibition of cell proliferation, migration and invasion, G2-M cell cycle arrest, and the induction of apoptosis in colon cancer cell lines [4]. Gaborit et al. reported that HER3 monoclonal antibody could inhibit the growth of gastric cancer cells (N87) *in vitro* and in animals [20]. Wu et al.'s study showed that knockdown of HER3 in the human gastric cancer cell line could inhibit cell proliferation and tumor growth both *in vitro* and *in vivo* by the inactivation of AKT [9].

Recent identification of several HER3 oncogenic mutations in colon and gastric cancers elucidated the role of HER3 in cancer development [6]. Jeong et al.'s study reported sporadic protein-altering HER3 mutations in 1% of colon cancers (1/100) [21]. Wang et al. observed that frequent mutations in the HER3 gene occurred in 10% of gastric cancers (2/22) [22]. A recent large-scale genomics study reported HER3 mutations in 7% of colon cancers (14/212) [23]. Jaiswal et al. reported the identification of HER3 somatic mutations in 11% of colon cancers (11/100) and 12% of gastric cancers (11/92). Jaiswal et al. also found that HER3 mutants co-expressed with HER2 promoted anchorage-independent growth of immortalized mouse colonic epithelial (IMCE) cells compared with HER3-wild type or the mutants when expressed on their own. Consistent with their ability to support anchorage-independent growth, IMCE cells co-expressing HER3 mutants along with HER2 showed an increase in tumor growth compared with HER3-wild type or HER2 alone or HER3-wild type and HER2 combined.

There are several potential limitations inherent to this meta-analysis. First, this is a literature-based analysis, and only published articles were included in this study; thus, publication bias may exist in this study. To address this issue, Egger's test and Begg's test were applied. Second, this study was based on population-level data rather than individual patient level data. Several confounders were not controlled, and multivariate analyses could not be conducted. Third, there is substantial heterogeneity in all of the analyses, which may not be completely interpreted by our use of the random-effects model. Fourth, the proportion of three years and five years survival was extracted directly from Kaplan-Meier curves, which may slightly deviate from the raw data.

## MATERIALS AND METHODS

### Literature and methods

A systematic search was performed using the Medline/PubMed, Embase, Science Direct and Elsevier databases with a combination of the terms: “HER3” or “ErbB3” or “human epidermal factor receptor 3” and “digestive tract cancer” or “gastrointestinal cancer” or “gastric cancer” or “colorectal cancer”. The ending date was July, 31, 2015.

The selection criteria included (1) the measurement of HER3 by immunohistochemistry; (2) the availability of survival data at three years and/or five years; and (3) the reporting of gastrointestinal cancers. Accordingly, papers without complete data, reviews and overlapping or duplicate papers were excluded.

**Figure 5:**
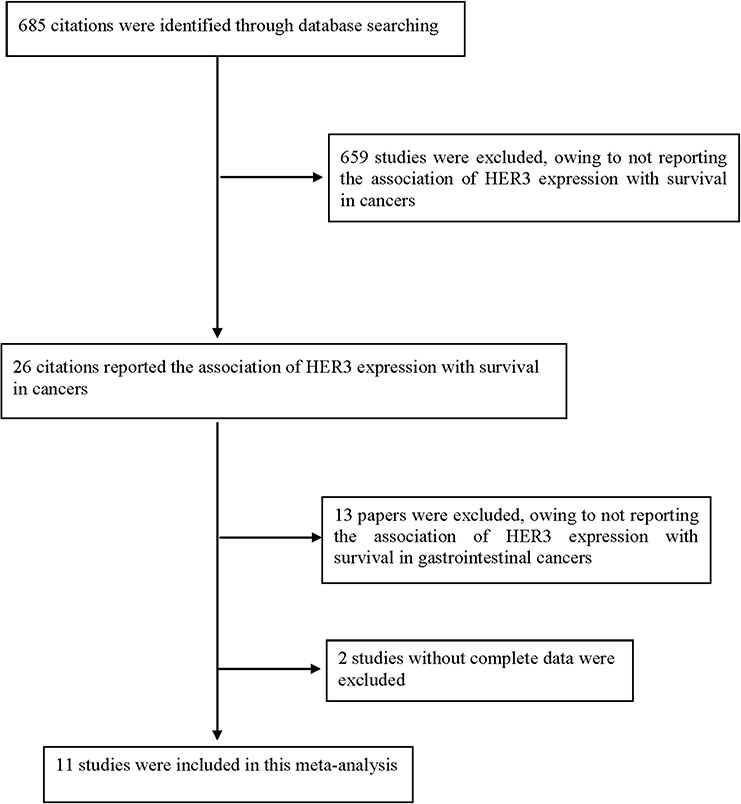
Flow diagram of the selection process

In total, thirteen published studies that reported an association between HER3 expression and overall survival in gastrointestinal cancers were identified. We reviewed all of these papers in accordance with the criteria defined above and excluded two papers [24, 25] without complete data. Therefore, eleven eligible studies were included in this study. The flow diagram of the selection process is shown in Fig. 5.

### Data extraction

Data were extracted and tabulated by two of the authors, and inputted into an electronic database independently. The following details were extracted: authors' name, year of publication, tumor type, number of patients, percentage of HER3 over-expression and cutoff of over-expression. The proportion of patients surviving to three and five years was extracted directly from the Kaplan-Meier curves. The quality of the studies was evaluated according to the Newcastle-Ottawa quality assessment scale [26, 27]. The main characteristics of individual studies are summarized in Table 2.

**Table 2:** Evaluation of HER3 by immunohistochemistry in the selected studies

Author	Year	Tumor Type	Number of patients	Percentage of HER3 over-expression (%)	Cutoff for HER3 over-expression	Quality score
Kountourakis [10]	2006	Colorectal	106	17.0%	Positive: Membranous staining: >1% of tumor cells stained. Cytoplasmic staining: 2+: moderate immunostaining in >10% of tumor cells and 3+: strong immunostaining in >10% of tumor cells.	6
Hayashi [11]	2008	Gastric	134	59.0%	Positive: 2+ = moderate staining, and 3+ = strong staining.	7
Baiocchi [12]	2009	Colorectal	109	69.7%	Cytoplasmic staining was scored as “0” with no staining or weak staining in <10% of tumor cells; membranous staining: “0” when there was no staining at all or membrane staining <10% of tumor cells; “1+” with perceptible membrane staining in >10% tumor cells; “2+” with weak-to-moderate staining of the entire membrane in more than 10% tumor cells; “3+” with strong staining of the entire membrane in >10% tumor cells.	5
Begnami [13]	2011	Gastric	191	34.0%	Slices with scores of 8 or higher were classified as positive, and slices with scores lower than 8 were classified as negative.	6
Beji [4]	2012	Colorectal	110	74.5%	HER3 expression was classified as weak, intermediate, or strong (analysis compared low vs. intermediate + strong).	6
Wu [9]	2014	Gastric	161	55.9%	Scores 0 and 1 were considered negative, and scores 2 and 3 were considered positive for HER3.	6
Ledel [15]	2014	Colorectal	236	69.5%	The intensity of staining was graded 0–3, where grade 0–1 was categorized as low, and grade 2–3 was categorized as high expression of membranous HER3.	7
Ema [14]	2014	Gastric	167	58.7%	0 = no staining observed in invasive tumor cells, 1+ = weak, incomplete membrane staining in any proportion of invasive tumor cells, or weak, complete membrane staining in less than 10% of cells, 2+ = complete membrane staining that is non-uniform or weak but with obvious circumferential distribution in at least 10% of cells, or intense complete membrane staining in 30% or less of tumor cells, 3+ = uniform intense membrane staining of more than 30% of invasive tumor cells.	6
Tang [16]	2015	Gastric	114	66.7%	0, if no staining was observed; 1+, if more than 10% of the tumor cells had weak staining on the membrane (or cytoplasm for HER3); 2+, if more than 10% of the tumor cells had moderate staining on the membrane (or cytoplasm for HER3); and 3+, if more than 10% of the tumor cells had strong staining on the membrane (or cytoplasm for HER3).	5
Seo [3]	2015	Colorectal	364	69.0%	0 and 1+ were considered no-over-expression, and 2+ and 3+ were considered over-expression.	7
He [17]	2015	Gastric	271	20.7%	Immunoreactive results of 2+ or 3+ were considered to be positive or high expression. By contrast, 0 or 1+ was evaluated as negative or low expression.	7

### Quantitative data synthesis

The heterogeneity among the studies was evaluated by the Cochrane Q statistics test. The random-effects model and fixed-effects model were used to estimate the summary ORs [28]. The fixed-effects model was used if the effects were assumed to be homogeneous; otherwise, the random-effects model was used. First, the publication bias was visually checked using funnel plots, and Begg's test and Egger's test were then used to further diagnose the publication bias [29, 30]. A sensitivity analysis was performed by deleting one study each time.

All of the statistical analyses were performed using STATA10.0 software package (Stata Corporation, College Station, TX, USA). All of the tests were two-sided, and a *P* value less than 0.05 was considered to be statistically significant.

## CONCLUSION

In summary, this meta-analysis found that HER3 over-expression was associated with worse overall survival at three years and five years in gastric cancers. Studies with a larger sample size are required to further verify our findings.
